# miR-27a Suppresses Mitochondrial Function to Promote Hepatic Steatosis in High-Fat-Diet-Induced Obesity

**DOI:** 10.3390/molecules31101753

**Published:** 2026-05-20

**Authors:** Zhiyi Yu, Xuehan Yang, Bin Sun, Yuhan Jiang, Yanfei Shi, Meishuang Zhang, Siwei Zhang, Fengying Guan

**Affiliations:** 1 Department of Pharmacology, College of Basic Medical Sciences, Jilin University, Changchun 130021, China; yzy18@mails.jlu.edu.cn (Z.Y.); hannahyang_11@outlook.com (X.Y.); sunbin23@mails.jlu.edu.cn (B.S.); yuhanj24@mails.jlu.edu.cn (Y.J.); shiyf25@mails.jlu.edu.cn (Y.S.); zhangmeishuang@jlu.edu.cn (M.Z.); 2 Department of Physiology and Pharmacology, Basic Medical College, Changchun Medical College, Changchun 130031, China; 3 Department of Rehabilitation, School of Nursing, Jilin University, Changchun 130021, China

**Keywords:** miR-27a, hepatic steatosis, NFE2L2, mitochondrial biogenesis, ROS

## Abstract

Non-coding RNAs are pivotal regulators of metabolic disease pathogenesis, yet the role of microRNA-27a (miR-27a) in obesity-associated hepatic steatosis remains incompletely characterized. This study examined the functional contribution and molecular mechanism of miR-27a in regulating hepatocyte mitochondrial homeostasis and lipid metabolism. Utilizing in vivo mouse models, including low-fat diet controls, high-fat diet (HFD)-induced obesity, and gain- and loss-of-function approaches, miR-27a was found to be markedly upregulated in the serum and liver of obese mice, correlating with disrupted glucose and lipid homeostasis as well as hepatic steatosis. Mechanistically, miR-27a overexpression recapitulated HFD-induced mitochondrial dysfunction, manifested by decreased mitochondrial biogenesis and elevated reactive oxygen species (ROS) production. Conversely, genetic silencing of miR-27a restored mitochondrial integrity and mitigated lipid accumulation. In vitro experiments using HepG2 cells confirmed that miR-27a directly suppresses nuclear factor erythroid 2-related factor 2 (NFE2L2), and NFE2L2 overexpression counteracted miR-27a-induced mitochondrial damage and steatosis. Collectively, these results demonstrate that miR-27a promotes hepatic steatosis by targeting NFE2L2, leading to mitochondrial impairment and oxidative stress, highlighting miR-27a as a potential biomarker and therapeutic target for obesity-associated liver metabolic disorders.

## 1. Introduction

Metabolic Dysfunction-Associated Steatotic Liver Disease (MASLD) affects approximately one-quarter of the global population, representing a major public health challenge due to its high prevalence, diagnostic complexity, and lack of definitive therapies [1]. The pathogenesis of MASLD is multifactorial, with the “two-hit” hypothesis traditionally proposed to explain disease progression: an initial hit driven by insulin resistance results in hepatic free fatty acid accumulation, while a subsequent hit involving oxidative stress and reactive oxygen species (ROS) overload exacerbates liver damage and steatosis [2,3]. Nevertheless, this sequential model is now considered overly simplistic. The contemporary “multiple parallel hits” hypothesis more accurately captures the pathophysiological complexity of MASLD, wherein insulin resistance, mitochondrial dysfunction, oxidative stress, inflammatory cascades, genetic and epigenetic factors, and gut microbiota alterations operate concurrently and interactively to promote disease initiation and progression [4]. Mitochondrial dysfunction—including impaired β-oxidation and reduced biogenesis—constitutes a central contributor to this process, as mitochondrial decline disrupts lipid metabolism and amplifies oxidative stress [3,5].

Mitochondrial biogenesis, the formation of new mitochondria coordinated by nuclear and mitochondrial genomes, is regulated by transcriptional cascades involving nuclear respiratory factor 1 (NRF1), mitochondrial transcription factor A (TFAM), and nuclear factor erythroid 2-related factor 2 (NFE2L2, Nrf2) [6,7,8]. NFE2L2 serves a dual role in hepatic homeostasis: it enhances mitochondrial biogenesis via upregulation of NRF1 and TFAM and functions as a central mediator of the antioxidant response to limit ROS accumulation [8,9,10]. Activation of NFE2L2 has been shown to ameliorate MASLD by promoting fatty acid oxidation and reducing oxidative stress [11,12,13]; however, the molecular factors responsible for downregulating NFE2L2 to facilitate hepatic steatosis remain incompletely defined.

To date, most studies have focused on NFE2L2 regulation by KEAP1, with limited insight into post-transcriptional mechanisms controlling its expression [9]. MicroRNAs (miRNAs)—endogenous ~20–24 nucleotide RNAs that repress gene expression post-transcriptionally—represent critical regulators of metabolic diseases, including insulin resistance, obesity, and MASLD [4]. Bioinformatic and experimental studies have identified miR-27a as a potential post-transcriptional regulator of NFE2L2 through direct binding to its 3′ untranslated region (3′UTR) [14,15]. Although miR-27a, released by adipose tissue, has been linked to systemic insulin resistance and obesity [16], its role in MASLD remains controversial and incompletely characterized [17,18,19].

In this study, it is hypothesized that miR-27a promotes hepatic steatosis by downregulating NFE2L2, thereby impairing mitochondrial biogenesis and increasing ROS accumulation. The objective is to elucidate the functional role and molecular mechanism of the miR-27a–NFE2L2 axis in obesity-associated hepatic steatosis, addressing critical gaps in MASLD pathogenesis and identifying potential therapeutic targets.

## 2. Results

### 2.1. Modulation of miR-27a Alleviates Hepatic Steatosis and Improves Glucose/Lipid Metabolism in Obese Mice

To investigate the effect of miR-27a on hepatic steatosis in mice, expression levels of miR-27a were modulated via lentivirus-mediated transduction. In low-fat diet (LFD)-fed mice, miR-27a was overexpressed, whereas in high-fat diet (HFD)-fed mice, miR-27a was knocked down using lentiviral shRNA.

**Figure 1:**
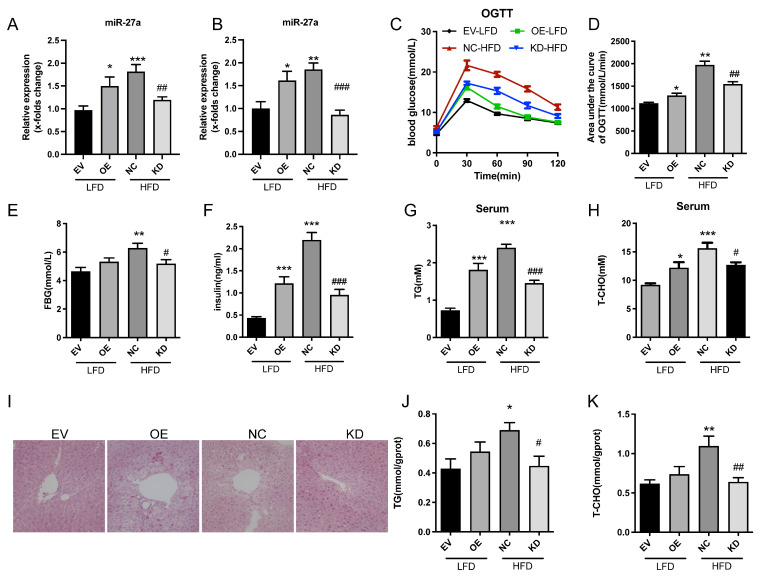
Modulation of miR-27a Alleviates Hepatic Steatosis and Improves Glucose/Lipid Metabolism in Obese Mice. (**A**,**B**) Quantitative analysis of miR-27a expression in serum (**A**) and liver tissues (**B**) of EV LFD, OE LFD, NC HFD, and KD HFD mice. N = 6. (**C**,**D**) OGTT curves (**C**) and corresponding area under the curve (AUC) analysis (**D**) in each group. N = 6. (**E**,**F**) FBG levels (**E**) and serum insulin concentrations (**F**) in indicated mice. N = 6. (**G**,**H**) Serum TG (**G**) and T-CHO (**H**) levels in each group. N = 6. (**I**) Histological analysis of liver sections by hematoxylin and eosin (HE) staining (scale bar = 100 μm). (**J**,**K**) Hepatic TG (**J**) and T-CHO (**K**) contents in liver tissues. N = 6. All data were analyzed by one-way ANOVA followed by Tukey’s post hoc test. * *p* < 0.05, ** *p* < 0.01, *** *p* < 0.001 versus EV LFD group; # *p* < 0.05, ## *p* < 0.01, ### *p* < 0.001 versus NC HFD group.

As shown in Figure 1A,B, both overexpression and knockdown of miR-27a were successfully achieved, as reflected by altered serum and liver expression. Notably, miR-27a levels were significantly elevated in HFD-fed mice, suggesting a correlation between miR-27a expression and obesity. To assess potential associations with insulin resistance, oral glucose tolerance tests (OGTT) were performed. Both HFD and miR-27a overexpression increased the area under the curve (AUC) of OGTT (Figure 1C,D), indicating impaired glucose tolerance and elevated insulin resistance. Conversely, miR-27a knockdown reduced OGTT AUC, suggesting partial amelioration of obesity-induced insulin resistance. Functionally, compared with LFD controls, fasting blood glucose (FBG), serum insulin (INS), total cholesterol (T-CHO), and triglyceride (TG) levels were significantly elevated in HFD-fed and miR-27a overexpressing mice (Figure 1E–H). Downregulation of miR-27a partially improved the glucose and lipid metabolic profile in HFD-induced obese mice. Histological analysis revealed that HFD feeding or miR-27a overexpression induced hepatocyte disorganization, nuclear displacement, and lipid droplet accumulation, leading to hepatic steatosis. In contrast, miR-27a knockdown attenuated tissue damage, with well-organized hepatocytes, uniform nuclei, and absence of visible lipid droplets, indicating significant improvement in liver morphology.

Consistent with these observations, hepatic TG and T-CHO levels were significantly higher in HFD and overexpression (OE) mice compared with LFD controls, whereas miR-27a silencing significantly reduced these lipid contents in obese mice (Figure 1J,K). Collectively, these findings support a role for miR-27a in the development of hepatic steatosis.

### 2.2. miR-27a Impairs Mitochondrial Function and Exacerbates Oxidative Damage in Hepatic Steatosis

Non-alcoholic steatohepatitis (NASH) is driven by progressive lipid toxicity, mitochondrial dysfunction, and unchecked oxidative stress, converging on the NFE2L2-dependent transcriptional network that governs mitochondrial biogenesis. To elucidate how miR-27a promotes liver injury in our diet-induced hepatic steatosis model, key mitochondrial parameters—including NFE2L2, TFAM, NRF1, and respiratory complexes III–V—were profiled to link defective mitochondrial renewal with elevated hepatic oxidative damage.

**Figure 2:**
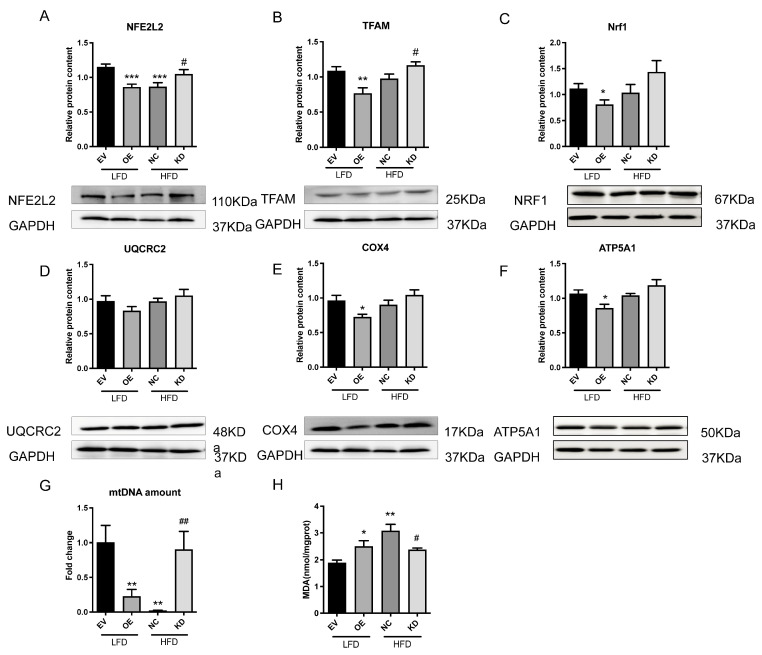
miR-27a Impairs Mitochondrial Function and Exacerbates Oxidative Damage in Hepatic Steatosis. (**A**–**F**) Representative Western blots and quantitative analysis of mitochondrial biogenesis-related proteins (NFE2L2, TFAM, NRF1) and mitochondrial respiratory chain complex subunits (UQCRC2, COX4, ATP5A1) in livers of indicated mice. (**G**) mtDNA content in livers of indicated mice. (**H**) Hepatic MDA content in indicated mice. All data were analyzed by one-way ANOVA followed by Tukey’s post hoc test. * *p* < 0.05, ** *p* < 0.01, *** *p* < 0.001 versus EV LFD group; # *p* < 0.05, ## *p* < 0.01 versus NC HFD group. N = 6 per group.

Compared with LFD-fed mice, NFE2L2 expression was markedly decreased in the livers of miR-27a overexpressing (OE) and HFD-fed mice (*p* < 0.001). miR-27a knockdown (KD) restored NFE2L2 levels to those observed in LFD controls (Figure 2A). Similarly, TFAM and NRF1 protein expression was significantly reduced in miR-27a OE and HFD mice, whereas KD mice exhibited upregulated TFAM and NRF1 expression relative to HFD controls (Figure 2B,C). Concomitant with NFE2L2 loss, TFAM and NRF1 protein levels were decreased in OE mice (both *p* < 0.01), and miR-27a knockdown reversed these reductions (*p* < 0.05 versus HFD, Figure 2B,C). Mitochondrial DNA (mtDNA) copy number mirrored these trends, decreasing in OE and HFD mice and recovering following miR-27a silencing (*p* < 0.01, Figure 2D). These data indicate that miR-27a overexpression impairs the mitochondrial biogenic program in mouse liver under MASLD conditions.

Having established that miR-27a overexpression curtails mitochondrial mass, the respiratory competence of the remaining organelles was assessed by quantifying the expression of complexes III, IV, and V. Immunoblotting revealed significant downregulation of complex III (UQCRC2), complex IV (COX4), and complex V (ATP5A1) in livers overexpressing miR-27a (all *p* < 0.05). These deficits were largely corrected by miR-27a knockdown in HFD mice (Figure 2D–F). These findings suggest that miR-27a overexpression downregulates mitochondrial complex protein expression, potentially reducing mitochondrial function. With mitochondrial biogenesis suppressed and respiratory chain integrity compromised, electron leakage and ROS generation were anticipated to increase. As expected, hepatic lipid peroxidation was quantified by measuring malondialdehyde (MDA).

Hepatic MDA content was elevated in miR-27a-overexpressing and HFD-fed mice (*p* < 0.05 and *p* < 0.01, respectively). Silencing of miR-27a restored MDA levels to those observed in LFD controls (*p* < 0.05 vs. HF, Figure 2H).

Collectively, these results indicate that miR-27a directly represses NFE2L2, thereby downregulating TFAM/NRF1-driven mitochondrial biogenesis and reducing respiratory chain complexes III–V. The consequent decrease in mitochondrial mass and respiratory efficiency amplifies ROS leakage, manifesting as increased hepatic lipid peroxidation and progressive liver injury.

### 2.3. miR-27a Suppresses Mitochondrial Function via Directly Targeting NFE2L2 in Hepatic Steatosis

**Figure 3:**
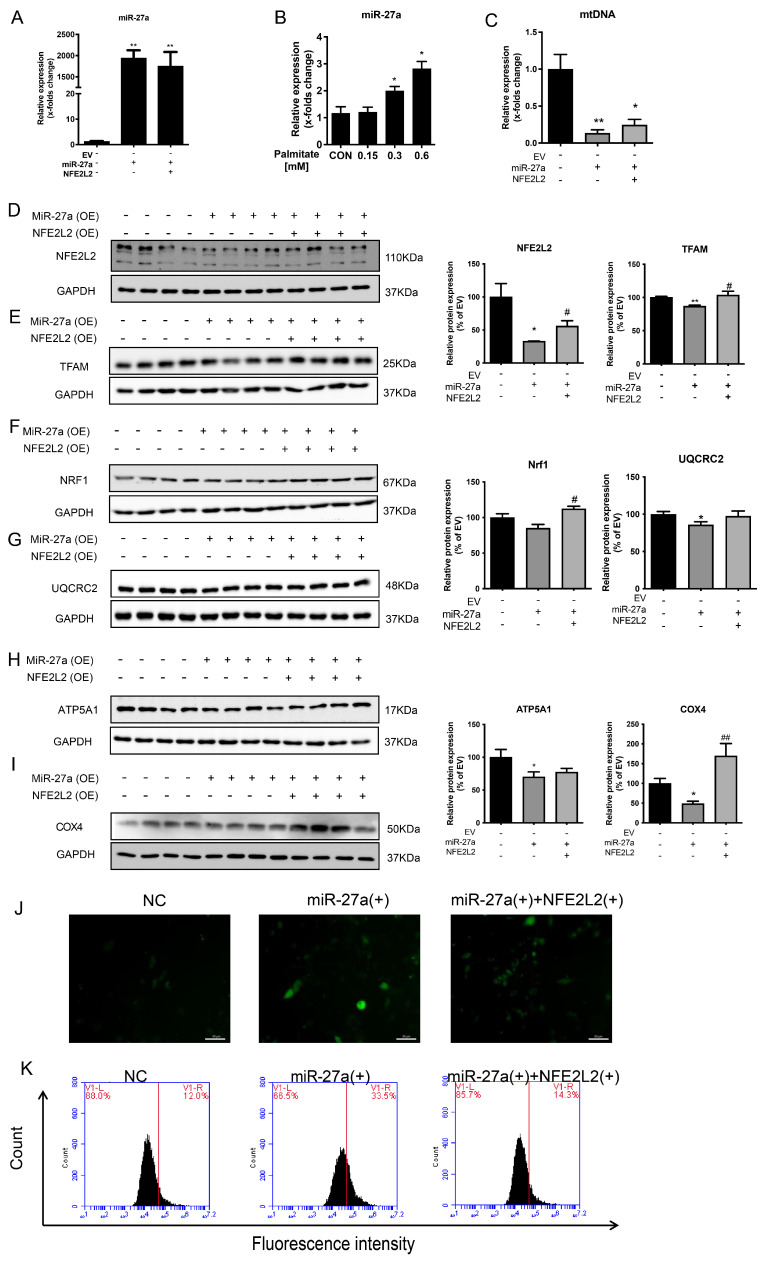
miR-27a Suppresses Mitochondrial Function via Directly Targeting NFE2L2 in Hepatic Steatosis. (**A**) Relative expression of miR-27a in HepG2 cells: control (NC), miR-27a overexpression (miR-27a(+)), and miR-27a/NFE2L2 co-overexpression (miR-27a(+)+NFE2L2(+)). (**B**) miR-27a expression in HepG2 cells treated with varying concentrations of palmitate (PA). (**C**) Relative mitochondrial DNA (mtDNA) content in HepG2 cells from indicated groups. (**D**–**I**) Representative Western blots and quantitative analysis of NFE2L2 (**D**), mitochondrial biogenesis regulators TFAM and NRF1 (**E**,**F**), and mitochondrial respiratory chain complex subunits UQCRC2, ATP5A1, and COX4 (**G**–**I**). GAPDH served as the loading control. (**J**) Representative fluorescence images of H_2_DCFDA staining for intracellular reactive oxygen species (ROS) in HepG2 cells. Scale bar = 50 μm. (**K**) Flow cytometry (FACS) analysis of H_2_DCFDA fluorescence intensity to quantify ROS levels in HepG2 cells. All data are presented as means ± SEM (*N* = 3 per group) and analyzed by one-way ANOVA followed by Tukey’s post hoc test. * *p* < 0.05, ** *p* < 0.01, versus NC/control group; # *p* < 0.05, ## *p* < 0.01 versus miR-27a(+) group.

To further elucidate the mechanism by which miR-27a regulates the development and progression of MASLD, a palmitate-induced intracellular lipid accumulation model was employed in HepG2 cells. miR-27a mRNA expression was markedly increased following miR-27a overexpression, confirming successful transfection. In contrast, NFE2L2 overexpression did not affect miR-27a levels (Figure 3A). Treatment with palmitate dose-dependently elevated miR-27a expression in HepG2 cells (Figure 3B), consistent with in vivo findings, suggesting that lipid accumulation in hepatocytes induces miR-27a expression.

Overexpression of miR-27a significantly decreased NFE2L2 protein expression in HepG2 cells (Figure 3C). Concurrently, mitochondrial biogenesis markers TFAM and NRF1 were reduced, accompanied by a substantial decline in mtDNA copy number, whereas NFE2L2 overexpression partially rescued these effects (Figure 3C–F).

Furthermore, miR-27a overexpression significantly downregulated mitochondrial complex III, IV, and V proteins, with NFE2L2 overexpression partially restoring COX4 expression (Figure 3G–I). H_2_DCFDA fluorescence imaging and flow cytometry revealed that miR-27a overexpression markedly increased intracellular ROS levels, which were attenuated upon NFE2L2 overexpression (Figure 3J,K). These results indicate that miR-27a functions upstream of NFE2L2 and suppresses mitochondrial function through direct targeting of this transcription factor.

### 2.4. miR-27a Disrupts Mitochondrial Morphology and Function via Targeting NFE2L2 in Hepatocytes

**Figure 4:**
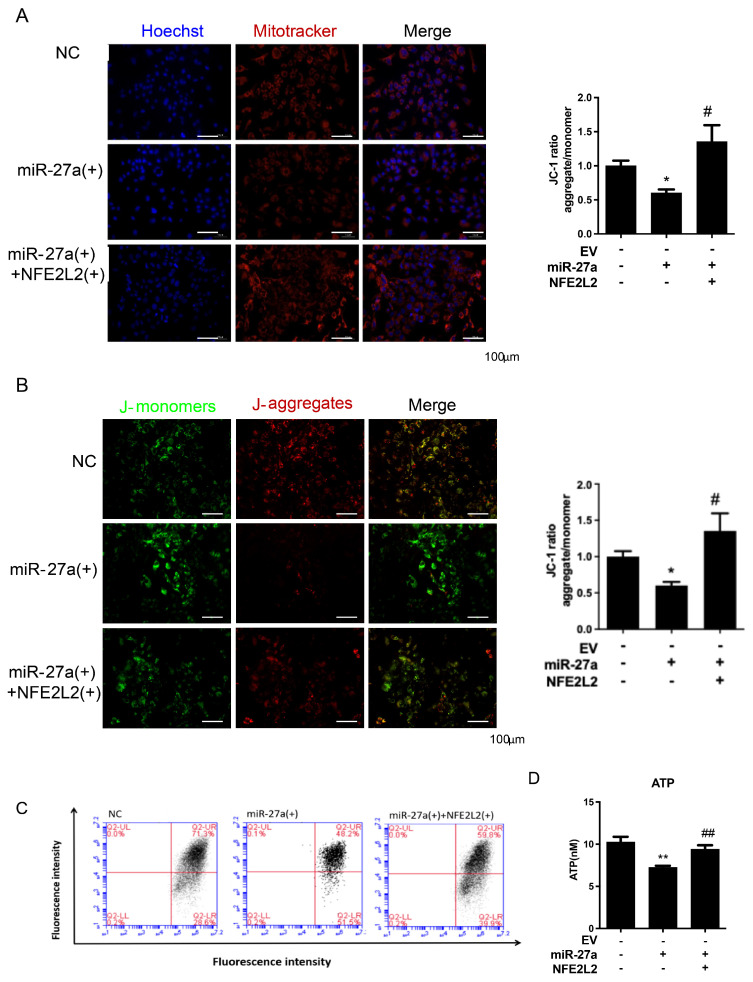
miR-27a Disrupts Mitochondrial Morphology and Function via Targeting NFE2L2 in Hepatocytes. (**A**) Representative immunofluorescence images of MitoTracker Red staining in HepG2 cells (NC, miR-27a(+), miR-27a(+)+NFE2L2(+)), with quantitative analysis of mean fluorescence intensity. Hoechst 33342 was used for nuclear counterstaining. Scale bar = 100 μm. (**B**) Representative immunofluorescence images of JC-1 staining in HepG2 cells, with quantitative analysis of the JC-1 aggregate/monomer ratio. Green fluorescence indicates JC-1 monomers (low mitochondrial membrane potential), and red fluorescence indicates JC-1 aggregates (high mitochondrial membrane potential). Scale bar = 100 μm. (**C**) Flow cytometry (FACS) analysis of JC-1 fluorescence intensity to quantify mitochondrial membrane potential. (**D**) Intracellular ATP levels in HepG2 cells of indicated groups. All data are presented as means ± SEM (N = 4 per group) and analyzed by one-way ANOVA followed by Tukey’s post hoc test. * *p* < 0.05, ** *p* < 0.01 versus NC group; # *p* < 0.05, ## *p* < 0.01 versus miR-27a(+) group.

MitoTracker Red (Beyotime, Shanghai, China) labeling revealed that the number of polarized mitochondria was reduced by miR-27a overexpression, an effect that was reversed by NFE2L2 co-overexpression (Figure 4A). Fluorescence microscopy and flow cytometry of JC-1 staining demonstrated a decrease in JC-1 aggregates and an increase in JC-1 monomers upon miR-27a overexpression, indicating a reduction in mitochondrial membrane potential. Consistently, intracellular ATP production was decreased in these cells. NFE2L2 overexpression effectively restored JC-1 aggregate formation and ATP levels (Figure 4B–D).

These results indicate that miR-27a, via downregulation of NFE2L2, compromises mitochondrial integrity, reduces mitochondrial membrane potential, and diminishes both the number and quality of polarized mitochondria, thereby impairing cellular energy production.

### 2.5. miR-27a Exacerbates Hepatic Lipid Accumulation by Suppressing NFE2L2

**Figure 5:**
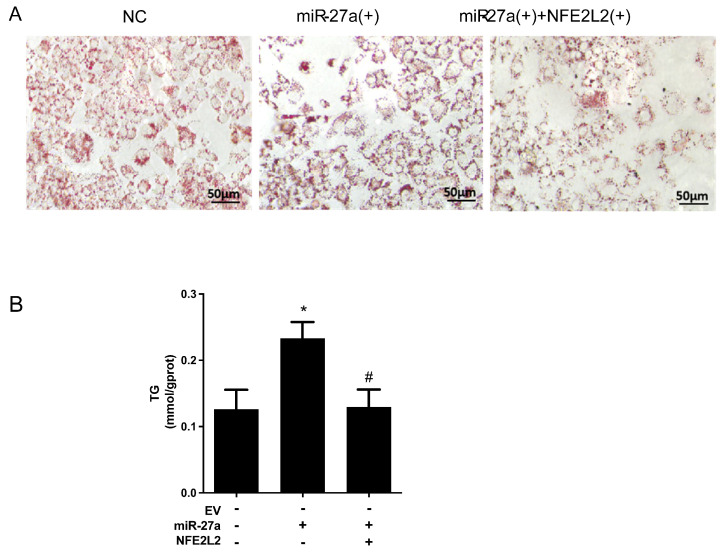
miR-27a Exacerbates Hepatic Lipid Accumulation by Suppressing NFE2L2. (**A**) Representative Oil Red O staining images of lipid droplets in HepG2 cells: CON (control), miR-27a(+), and miR-27a(+)+NFE2L2(+) groups. Scale bar = 50 μm. (**B**) Intracellular triglyceride (TG) content in HepG2 cells of indicated groups. All data are presented as means ± SEM (N = 3 per group) and analyzed by one-way ANOVA followed by Tukey’s post hoc test. * *p* < 0.05 versus CON/control group; # *p* < 0.05 versus miR-27a(+) group.

Having demonstrated that lipid overload upregulates miR-27a, the next question was whether miR-27a itself could drive steatosis. Overexpression of miR-27a alone in HepG2 cells was sufficient to induce substantial lipid accumulation: Oil Red O staining revealed a pronounced increase in both the number and size of cytoplasmic lipid droplets, and cellular TG content was elevated accordingly (Figure 5A,B). Co-overexpression of NFE2L2 almost completely reversed these changes, confirming that miR-27a promotes hepatic steatosis by silencing NFE2L2.

**Figure 6:**
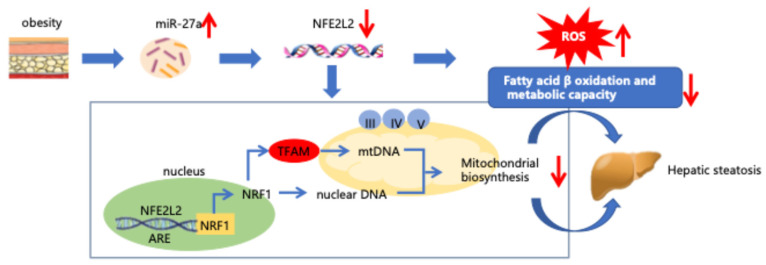
Schematics of proposed mechanism.

In obesity (Figure 6), upregulated miR-27a directly targets and suppresses the transcription factor NFE2L2 in hepatocytes. This downregulation disrupts two essential homeostatic pathways: mitochondrial biogenesis—reflected by reduced expression of NRF1, TFAM, and mitochondrial respiratory chain complexes III, IV, and V—and antioxidant defense. The resulting impairment decreases fatty acid β-oxidation, elevates ROS accumulation, and establishes a vicious cycle of oxidative stress and metabolic dysfunction. Collectively, these disturbances promote hepatic lipid accumulation and drive the development of MASLD.

## 3. Discussion

MASLD represents a global health burden with no definitive therapeutic strategies, highlighting the urgent need to elucidate its complex pathogenesis and identify actionable molecular targets. miRNAs have emerged as critical post-transcriptional regulators of hepatic metabolism, orchestrating lipid anabolism, catabolism, and transport, as well as modulating carbohydrate metabolism and stress-response pathways that contribute to steatosis development [4]. Among these, miR-122, miR-33, and miR-21 have been implicated in the initiation and progression of MASLD, highlighting the potential of miRNAs as both diagnostic biomarkers and therapeutic candidates [20,21,22]. However, the role of miR-27a in MASLD pathogenesis remains incompletely defined, representing a critical gap in understanding miRNA-mediated regulation of hepatic lipid homeostasis.

Our results establish miR-27a as a key driver of hepatic steatosis in vivo. Within the framework of the multiple parallel hits hypothesis, the miR-27a–NFE2L2 axis emerges as an integrative molecular node that concurrently impairs mitochondrial biogenesis and antioxidant defense, thereby linking two distinct pathogenic hits—metabolic overload and oxidative stress—into a unified pro-steatotic signaling pathway. Lentiviral overexpression of miR-27a in LFD-fed mice was sufficient to induce hallmark MASLD features, including systemic insulin resistance, hepatic lipid vacuole accumulation, and elevated TG and cholesterol levels in both liver and serum. Conversely, silencing miR-27a in HFD-fed obese mice improved insulin sensitivity, normalized lipid profiles, and alleviated hepatic steatosis. These reciprocal gain- and loss-of-function experiments support a causal role for miR-27a in diet-induced hepatic steatosis, rather than a mere correlative association as reported for other metabolic miRNAs [20,21,22].

Circulating and tissue-resident miRNAs have been recognized as critical modulators of systemic metabolism, with exosomal miRNAs particularly implicated in coordinating lipid and glucose homeostasis [23]. Adipose-derived miR-27a has been previously linked to obesity-induced insulin resistance in skeletal muscle [16], supporting its role as a systemic metabolic signal. In our study, palmitic acid treatment dose-dependently increased miR-27a expression in HepG2 cells, suggesting that miR-27a originates from both adipose tissue secretion and hepatic lipid-induced upregulation during obesity. These dual sources likely converge to promote hepatic steatosis.

As a canonical post-transcriptional repressor, miR-27a has been bioinformatically and experimentally validated to directly target the 3′UTR of *NFE2L2* [14,15,24]. Consistent with these findings, our in vivo data demonstrated that miR-27a overexpression suppressed hepatic NFE2L2 protein levels, whereas miR-27a knockdown restored NFE2L2 expression in obese mice. Given that NFE2L2 activation protects against lipid accumulation and MASLD [11,12,13], these results support a model in which miR-27a drives hepatic steatosis, at least in part, by silencing NFE2L2.

NFE2L2 is a central regulator of the cellular antioxidant response, mitigating oxidative damage by reducing ROS levels and inducing cytoprotective gene expression [25]. Excessive ROS promotes oxidative stress, disrupts metabolic homeostasis, and directly damages mtDNA [26]. Consistent with this, hepatic MDA levels—a marker of lipid peroxidation—were elevated in miR-27a-overexpressing mice, whereas miR-27a silencing reduced oxidative stress in obese animals. These findings reinforce a functional link between miR-27a, NFE2L2 suppression, and enhanced hepatic oxidative stress.

Beyond its role in antioxidant defense, NFE2L2 governs mitochondrial biogenesis by transcriptionally regulating *Nrf1* and *Tfam* [27], key factors required for mtDNA replication and maintenance of the mitochondrial network [28,29]. Notably, miR-27a has been previously implicated in repressing mitochondrial biogenesis through alternative targets [30]. For example, Giovannina Barisciano et al. demonstrated that the miR-27a/FOXJ3 axis downregulates mitochondrial biogenesis [31]. Our data extend these observations, showing that miR-27a overexpression reduced levels of mitochondrial respiratory chain complexes III, IV, and V, as well as cellular ATP content, indicative of impaired oxidative phosphorylation and mitochondrial dysfunction. Conversely, inhibiting miR-27a in obese mice restored mitochondrial biogenesis and function.

Specifically, miR-27a overexpression decreased the expression of mitochondrial complexes III, IV, and V, along with ATP content. This decline may result from oxidative stress-induced mitochondrial damage, reducing the activity of complexes III and IV and thereby decreasing the rate of oxidative phosphorylation. As complex V functions as a rate-limiting enzyme in ATP synthesis, its reduced expression corresponds with the observed ATP decline. These findings are consistent with prior studies in the heart, where decreased mitochondrial biogenesis was associated with diminished activity of complexes I–III and V [32], and may similarly reflect liver mitochondrial abnormalities involving low levels of complexes I, III, IV, and V. Collectively, these results suggest that miR-27a-mediated downregulation of NFE2L2 leads to mitochondrial dysfunction.

Overall, our findings establish that miR-27a-mediated suppression of NFE2L2 triggers dual pathological insults—elevated oxidative stress and impaired mitochondrial biogenesis—that synergistically disrupt hepatic lipid handling and promote steatosis. By linking miR-27a to NFE2L2-dependent mitochondrial homeostasis, this study uncovers a previously unrecognized regulatory axis in MASLD pathogenesis.

It is noteworthy that NFE2L2 overexpression nearly completely reversed miR-27a-induced hepatic lipid accumulation in HepG2 cells (Figure 5), yet only partially restored mitochondrial biogenesis markers, respiratory chain complex expression, and ATP production (Figure 3 and Figure 4). This divergence indicates that the miR-27a/NFE2L2 axis, although critical, may function within a broader regulatory network encompassing additional parallel pathways. For example, miR-27a has been reported to target PPARγ in skeletal muscle insulin resistance and FOXJ3 in the mitochondrial homeostasis of other cell types, suggesting that NFE2L2-independent branches may contribute to the full mitochondrial and metabolic phenotype. Future studies employing multi-omics approaches or epistasis analyses will be necessary to delineate the relative contributions of NFE2L2-dependent versus NFE2L2-independent pathways downstream of miR-27a in hepatocytes.

Several limitations should be acknowledged. Mouse models may incompletely recapitulate human metabolic genetics, environmental exposures, and gut microbiota influences. The animal experiments were conducted as two parallel gain- and loss-of-function studies under distinct dietary conditions rather than a full 2 × 2 factorial design; thus, potential interactions between diet and miR-27a levels were not formally assessed. Furthermore, the relatively short intervention period does not fully emulate chronic human MASLD progression. The use of HepG2 cells, a human hepatoma cell line, represents an additional limitation, as their metabolism substantially differs from that of healthy primary hepatocytes. HepG2 cells exhibit altered mitochondrial oxidative capacity, enhanced glycolytic flux, and constitutively activated oncogenic signaling pathways, which may confound the interpretation of lipid metabolism and stress responses. Consequently, the in vitro findings should be interpreted with caution and require validation in primary mouse or human hepatocytes, as well as in non-transformed hepatic cell lines, to confirm the generalizability of the miR-27a/NFE2L2 axis under physiological conditions.

## 4. Materials and Methods

### 4.1. Animals

Male C57BL/6J mice, aged three weeks (HFK Bioscience, Beijing, China), were housed under standard conditions with controlled humidity (55 ± 5%) and temperature (22 ± 2 °C), following a 12 h light–dark cycle, for one week to acclimatize. Mice were then assigned to either an HFD (D12492, Research Diets, New Brunswick, NJ, USA) or an LFD (10% kcal fat, D12450B, Research Diets, New Brunswick, NJ, USA).

After four weeks, the LFD group was randomly divided into two subgroups: the EV group, receiving lentivirus with an empty vector (low-fat-fed, n = 6) as the control, and the OE group, receiving a tail vein injection of miR-27a overexpressing lentivirus (100 µL, 5 × 10^7^ TU/mL, Genechem, Shanghai, China, n = 6).

Similarly, the HFD group was subdivided into the NC group, injected with lentivirus expressing a non-targeting control (n = 6), and the KD group, subjected to miR-27a knockdown via lentivirus (100 µL, 5 × 10^7^ TU/mL, Genechem, Shanghai, China, n = 6). Lentiviral vectors for miR-27a overexpression and knockdown were driven by the CMV promoter (for the miR-27a precursor expression cassette) and the U6 promoter (for the shRNA expression cassette), respectively.

Following a 12-week dietary intervention, mice underwent fasting plasma glucose measurement and an OGTT. Mice were deeply anesthetized with sodium pentobarbital (150 mg/kg, i.p.) for complete blood collection. Whole blood was centrifuged at 3500 rpm for 15 min at 4 °C to obtain serum, which was stored for subsequent analyses. Mice were then euthanized by an additional overdose of sodium pentobarbital, confirmed by absence of toe-pinch reflex and respiration, and livers were extracted. Liver tissues were preserved in 10% formalin for histopathology and at −80 °C for protein analyses. Comparison with the LFD group indicated that the control vector group showed no significant differences in body weight, miR-27a levels, insulin, or blood lipid profiles (Figure S1).

All animal procedures conformed to the Guide for the Care and Use of Laboratory Animals and were approved by the Institutional Animal Care and Use Committee of Jilin University (permit number: SYXK 2019-0015).

### 4.2. Oral Glucose Tolerance Test

For OGTT, mice were fasted for 12 h overnight and orally administered 2 g/kg body weight of 40% water-soluble glucose. Blood glucose was measured at 0, 30, 60, 90, and 120 min using a glucometer (Roche, South San Francisco, CA, USA) with test strips (Roche, Basel, Switzerland) after 10 µL of blood was obtained from the tail tip. The area under the glucose curve was subsequently calculated.

### 4.3. Serum Biochemical Parameters

At the end of the experimental period, mice were fasted for 12 h prior to blood collection from the medial canthus vein. After incubation at 37 °C for 15 min, sera were separated by centrifugation (3500 rpm, 15 min, 4 °C). Serum TG and T-CHO levels were measured using commercial kits (Nanjing Jiancheng, China), and fasting insulin concentrations were determined by ELISA (ALPCO, Salem, NH, USA).

#### Preparation of Liver Homogenates and Determination of Liver TC and T-CHO

Liver homogenates were prepared as previously described [33]. Briefly, fresh liver tissues were rinsed with ice-cold phosphate-buffered saline (PBS, pH 7.4) to remove residual blood, blotted dry, and weighed. Tissues were homogenized in ice-cold PBS (1:9, *w*/*v*) using a tissue homogenizer at 800 rpm for 30 s, with all procedures performed on ice to prevent protein denaturation. The homogenate was centrifuged at 3000 rpm for 15 min at 4 °C, and the supernatant was collected.

Protein concentrations were determined using a BCA assay [34]. TG and T-CHO levels were measured using commercial kits (Nanjing Jiancheng, China).

### 4.4. Liver Histopathology

Following euthanasia by sodium pentobarbital overdose, livers were immediately excised. Residual blood was aspirated from the tissue, which was then fixed in 4% paraformaldehyde (PFA) for 24 h at room temperature.

#### 4.4.1. Tissue Processing and Sectioning

Fixed tissues were thoroughly washed with cold PBS to remove residual fixative. Tissues were dehydrated through a graded ethanol series (70%, 80%, 90%, 95%, and 100%), cleared in xylene, and embedded in paraffin wax. Sections of 5 µm thickness were cut using a microtome, mounted on glass slides, and dried overnight at 37 °C.

#### 4.4.2. Histological Staining and Microscopy

Paraffin sections were dewaxed with xylene and rehydrated through a descending ethanol series (100%, 95%, 90%, 80%, and 70%) to distilled water. Sections were stained with hematoxylin for 5 min, differentiated with 1% acid alcohol for 30 s, rinsed, and counterstained with eosin for 2 min. Stained sections were dehydrated through an ascending ethanol series, cleared in xylene, and mounted with neutral gum. Images were captured using a DP73 camera attached to an Olympus BX53 microscope (Tokyo, Japan).

#### 4.4.3. Assessment of Malondialdehyde (MDA) Content

MDA contents were determined using a commercial kit (Nanjing Jiancheng, China) according to the manufacturer’s instructions. A portion of liver tissue was used to prepare a 10% tissue homogenate without centrifugation. The homogenate was thoroughly mixed in a 15 mL centrifuge tube. Reagents and samples were added according to kit instructions, mixed, and 200 µL of the mixture was transferred to a 96-well plate. Absorbance was measured, and MDA content was calculated.

#### 4.4.4. Cell Culture and Treatment

Human hepatoma (HepG2) cells, obtained from Genetic Testing Biotechnology (Suzhou, China), were maintained in a humidified incubator at 37 °C with 5% CO_2_. Cells were cultured in Dulbecco’s Modified Eagle’s Medium (DMEM) containing 4.5 g/L glucose, supplemented with 10% fetal bovine serum (FBS) and 1% penicillin/streptomycin (Sigma-Aldrich, St. Louis, MO, USA). Media were replaced or cells subcultured every other day. Transfection experiments were conducted when cell density reached approximately 70%.

#### 4.4.5. Plasmid Transduction and Expansion

Lyophilized plasmids expressing hsa-miR-27a-3p mimic and NFE2L2 were obtained from GenePharma (Suzhou, China). Plasmids were resuspended in distilled water and heat-transformed into chemically competent *E. coli* (DH5α). After expansion in LB medium containing appropriate antibiotics, plasmids were isolated using a commercial plasmid maxiprep kit (BIO TEK, Winooski, VT, USA).

#### 4.4.6. Plasmid Transfection

Plasmid transfection was performed using Lipofectamine 2000 (Invitrogen, San Diego, CA, USA) according to the manufacturer’s protocol. HepG2 cells were seeded 24 h prior to transfection. Briefly, 2.5 µg of plasmid (miR-27a mimic and/or NFE2L2 plasmid) and 8 µL of Lipofectamine 2000 were each diluted in 200 µL of serum-free Opti-MEM medium (Gibco). After 5 min, the plasmid and Lipofectamine solutions were combined at a 1:1 ratio and incubated at room temperature for 15 min. The resulting complexes were added to the cells. Experimental groups included: CON (control), miR-27a(+) (miR-27a overexpression), and miR-27a(+) + NFE2L2(+) (simultaneous overexpression of miR-27a and NFE2L2).

#### 4.4.7. Luciferase Reporter Assay

A luciferase reporter assay was performed as previously described [35,36]. The human NFE2L2 3′ UTR containing the predicted hsa-miR-27a-3p target site was amplified from genomic DNA by PCR and inserted into the pGL6-CMV-Luc vector (D2091, Beyotime, Shanghai, China), which encodes both firefly and Renilla luciferases. Cells were co-transfected with the wild-type reporter plasmid and miRNA mimic using Lipofectamine 2000. Luciferase activity was measured 48 h post-transfection using a Gaussia Luciferase Assay Kit (RG021S, Beyotime, Shanghai, China), and firefly luciferase activity was normalized to *Renilla luciferase* activity.

#### 4.4.8. In Vitro MASLD Induction and Oil Red O Staining

To induce MASLD in vitro, sodium palmitate and sodium oleate were first complexed with bovine serum albumin (BSA). Sodium palmitate was dissolved in ultrapure water at 70 °C and slowly added to a pre-warmed 20% (*w*/*v*) BSA solution (maintained at 70 °C) under constant stirring to yield a 7.5 mM stock. Sodium oleate was dissolved at 75 °C and complexed with 20% BSA to obtain a 10 mM stock. Fatty acid–BSA complexes were sterile-filtered (0.22 µm) and stored at 4 °C. Six hours after transfection, media were replaced with fresh high-glucose DMEM containing 25 µM palmitic acid (PA) and 50 µM oleic acid (OA) from the BSA-conjugated stocks. The 42 h free fatty acid exposure (initiated 6 h post-transfection) was based on pilot studies and published protocols indicating that 24–48 h of PA/OA treatment induces robust hepatic steatosis in HepG2 cells while maintaining cell viability. After 42 h, supernatants were discarded, cells were washed with PBS, and fixed with 4% PFA for 30 min. Cells were washed with PBS for 10 min and stained with 1.5 mL of Oil Red O working solution for 30 min. Excess stain was removed by rinsing 2–3 times with ultrapure water, and images were captured using an Olympus BX53 microscope (Tokyo, Japan).

#### 4.4.9. MitoTracker Red Staining

For mitochondrial and nuclear staining, cells transfected with the indicated plasmids were seeded on coverslips in 6-well plates. Cells were incubated with 1 µM MitoTracker Red in growth medium at 37 °C for 30 min, followed by Hoechst 33258 (0.5–10 µg/mL) for 5 min. Coverslips were washed with PBS, mounted on glass slides, and imaged. Fluorescence intensity was quantified using ImageJ software (v1.43).

#### 4.4.10. ROS Detection by H_2_DCFDA (DCFH-DA) Staining

Cells transfected with the indicated plasmids were seeded on coverslips or in 6-well plates. For ROS detection, cells were incubated with 5 µM H_2_DCFDA in PBS at 37 °C for 30 min. Coverslips were then mounted on glass slides for imaging using an Axio Imager Z2 fluorescence microscope (Zeiss, Oberkochen, Germany).

Cells cultured in 6-well plates were harvested, and ROS levels were measured using an Accuri C6 Flow Cytometer (BD, Franklin Lakes, NJ, USA).

#### 4.4.11. Mitochondrial Membrane Potential Assessment by JC-1 Staining

Cells transfected with indicated plasmids were seeded on coverslips or in 6-well plates.

Mitochondrial membrane potential (ΔΨm) was assessed using a JC-1 staining kit (Beyotime, Shanghai, China). Cells were washed twice with pre-warmed PBS and incubated with JC-1 working solution for 20 min at 37 °C.

Following two washes with JC-1 staining buffer, coverslips were mounted on slides for fluorescence imaging (Zeiss, Oberkochen, Germany), and fluorescence intensity was quantified using ImageJ software (v1.43). Cells in 6-well plates were harvested, and JC-1 fluorescence was measured by flow cytometry using an Accuri C6 Flow Cytometer (BD, Franklin Lakes, NJ, USA).

#### 4.4.12. Total ATP Content

Cellular ATP content was determined using a luciferase-based ATP Assay Kit (Beyotime, Shanghai, China). Transfected cells were rinsed with PBS, lysed, and centrifuged at 12,000× *g* for 5 min at 4 °C. The supernatant was collected, and 100 µL of ATP detection solution was added to a black 96-well plate and incubated for 3–5 min at room temperature. Subsequently, 20 µL of ATP standard or sample was added to each well, and relative luminescence units (RLU) were measured using a BioTek Synergy H1 microplate reader (BioTek, Winooski, VT, USA). Protein concentrations were determined using a Bradford Assay Kit. Cellular ATP content was calculated as normalized RLU (NRLU; nmol/mg protein).

#### 4.4.13. Mitochondrial DNA Copy Number

Mitochondrial density was evaluated via qPCR analysis of mtDNA. Total DNA was extracted from liver tissue or HepG2 cells using the Blood/Cell/Tissue Genomic DNA Extraction Kit (Tiangen, Beijing, China) according to the manufacturer’s instructions. In liver tissue, the relative mitochondrial copy number was calculated as the ratio of the mitochondrial gene (mtDNA) to the nuclear gene RBM15, representing mtDNA and nuclear DNA (nDNA) copy numbers, respectively. In HepG2 cells, the mtDNA copy number was determined as the ratio of Nuclear18s rRNA (a nuclear-encoded gene) to Cytochrome b (a mitochondrial-encoded gene).

#### 4.4.14. Real-Time Quantitative PCR

Total RNA was extracted from liver tissue and HepG2 cells using TRIzol reagent (Thermo Fisher Scientific, Rockford, IL, USA).

First-strand cDNA synthesis was performed using a reverse transcription kit (TransGen Biotech, Beijing, China) according to the manufacturer’s protocol.

Real-time quantitative PCR was conducted on a QuantStudio 3 RT–PCR instrument (Thermo Fisher Scientific, Rockford, IL, USA) using Fast Start Universal SYBR Green Master Mix (Roche, South San Francisco, CA, USA).

The relative expression levels compared to those in the control group were determined by calculating the 2^−ΔΔCT^ method [37].

For miR-27a quantification, U6 served as the reference gene. PCR primers were synthesized by RiboBio (Guangzhou, China), and sequences are listed in Table 1. Reference sequences for miR-27a, U6 [16], human Cytochrome b–mtDNA [38], human Nuclear 18s rRNA [39], and mouse mtDNA and RBM15 [40] were used.

#### 4.4.15. Western Blot Analysis

Total proteins were extracted from HepG2 cells and liver tissue using RIPA buffer (Invent, Eden Prairie, MN, USA). Protein concentrations were determined with the BCA protein assay kit (Thermo Fisher Scientific, Rockford, IL, USA).

Equal amounts of protein were loaded and separated on 12% or 15% SDS–polyacrylamide gels, followed by transfer onto PVDF membranes (Bio-Rad, USA) using a tank blot system (200 mA, 90 min). Membranes were blocked with 5% (w/v) BSA in TBS-T for 1 h at room temperature and subsequently incubated with primary antibodies at 4 °C overnight with gentle agitation. The primary antibodies employed included mouse anti-ATP5A1 (1:5000, Proteintech, Rosemont, IL, USA), rabbit anti-GAPDH (1:2000, Proteintech, Rosemont, IL, USA), rabbit anti-COX-4 (1:1000, Cell Signaling Technology, Danvers, MA, USA), rabbit anti-NFE2L2 (1:1000, Proteintech, Rosemont, IL, USA), NRF1 (1:2000, Proteintech, Rosemont, IL, USA), TFAM (1:1000, Proteintech, Rosemont, IL, USA), and rabbit anti-UQCRC2 (1:2000, Proteintech, Rosemont, IL, USA). Following three 10 min washes with TBST, membranes were incubated with horseradish peroxidase-conjugated secondary antibodies (goat anti-mouse or goat anti-rabbit) at a 1:5000 dilution (Proteintech, Rosemont, IL, USA) for 2 h at room temperature. After three additional washes with TBST, membranes were incubated with ECL developer solution (Thermo Fisher Scientific, Rockford, IL, USA) for 2 min, and chemiluminescence was detected using a gel imaging system (Biotek, Winooski, VT, USA). Densitometric analysis was conducted using Quantity One software (v4.6.2).

#### 4.4.16. Statistical Analysis

Data are expressed as means ± S.E.M. Statistical analyses were performed using GraphPad Prism 7 software (GraphPad Software, San Diego, CA, USA).

Sample size calculations were conducted in GraphPad Prism 7. A priori power analysis for one-way ANOVA was performed with four groups, α = 0.05, desired power (1 − β) = 0.80, and expected effect sizes derived from published hepatic steatosis studies and preliminary observations. This analysis assumed a minimum detectable difference of approximately 30–35% in primary outcomes (e.g., hepatic TG content and OGTT-AUC) with an estimated pooled standard deviation from prior literature using comparable HFD models. The calculation indicated that n = 5–6 per group was required to achieve 80% power; therefore, n = 6 per group was used to account for potential biological variability.

For cell-based experiments, a sample size of n = 3 was selected based on resource availability and the anticipated large effect size from preliminary data.

Comparisons across multiple groups were performed using one-way analysis of variance (ANOVA) followed by Tukey’s post hoc test for pairwise comparisons against the control group.

## 5. Conclusions

In conclusion, the miR-27a–NFE2L2 axis was identified as a critical driver of hepatic steatosis, mediating its effects through impaired mitochondrial biogenesis and elevated oxidative stress. These findings enhance the mechanistic understanding of MASLD and support the rationale for targeting miR-27a as a potential therapeutic strategy to prevent or reverse early-stage liver disease.

## Figures and Tables

**Table 1:** Primer sequences.

Primer Name	Sequences (5′-3p)
miR-27a-F	5′-TGCGCTTCACAGTGGCTAAGT-3′
miR-27a-R	5′-CCAGTGCAGGGTCCGAGGTATT-3′
U6-F	5′-CGCTTCGGCAGCACATATAC-3′
U6-R	5′-AAATATGGAACGCTTCACGA-3′
homo-Cytochrome b-mtDNA-F	5′- AACTTCGGCTCACTCCTTGG-3′
homo-Cytochrome b-mtDNA-R	5′- CCAATGTATGGGATGGCGGA-3′
homo-Nuclear 18s rRNA-F	5′-ACGGACCAGAGCGAAAGCA-3′
homo-Nuclear 18s rRNA-R	5′-GACATCTAAGGGCATCACAGAC-3′
mous-mtDNA-F	5′-AGGAGCCTGTTCTATAATCGATAAA-3′
mous-mtDNA-R	5′-GATGGCGGTATATAGGCCGAA-3′
mous-RBM15-F	5′-GGACAGTTTTCTTGGGCAAC-3′
mous-RBM15-R	5′-AGTTTGGCCCTGTGAGACAT-3′

## Data Availability

The datasets used and/or analyzed during the current study are available from the corresponding author on reasonable request.

## Supplementary Materials

The following supporting information can be downloaded at: https://www.mdpi.com/article/10.3390/molecules31101753/s1.

## Abbreviations

The following abbreviations are used in this manuscript:

ATP	Adenosine Triphosphate
ATP5A1	ATP Synthase F1 Subunit Alpha
AUC	Area Under the Curve
BCA	Bicinchoninic Acid
BSA	Bovine Serum Albumin
COX4	Cytochrome c Oxidase Subunit 4
FACS	Fluorescence-Activated Cell Sorting
FBG	Fasting Blood Glucose
H_2_DCFDA	2′,7′-Dichlorodihydrofluorescein Diacetate
INS	Insulin
MDA	Malondialdehyde
MASLD	Metabolic Dysfunction-Associated Steatotic Liver Disease
NC	Negative Control
NFE2L2	Nuclear Factor, Erythroid 2 Like 2
NRF1	Nuclear Respiratory Factor 1
NRLU	Normalized Relative Light Unit
OA	Oleic Acid
OGTT	Oral Glucose Tolerance Test
PA	Palmitic Acid
PCR	Polymerase Chain Reaction
PFA	Paraformaldehyde
RBM15	RNA Binding Motif Protein 15
RLU	Relative Light Unit
SDS	Sodium Dodecyl Sulfate
TFAM	Mitochondrial Transcription Factor A
TG	Triglyceride
UTR	Untranslated Region
