# Supplementary material for: miR-27a Suppresses Mitochondrial Function to Promote Hepatic Steatosis in High-Fat-Diet-Induced Obesity
Source: Molecules. 2026 May 20;31(10):1753. doi: 10.3390/molecules31101753 (PMC13209732; doi:10.3390/molecules31101753)
Supplement: Supplementary file 1 [file molecules-31-01753-s001.zip › molecules-4301753-supplementary.pdf]

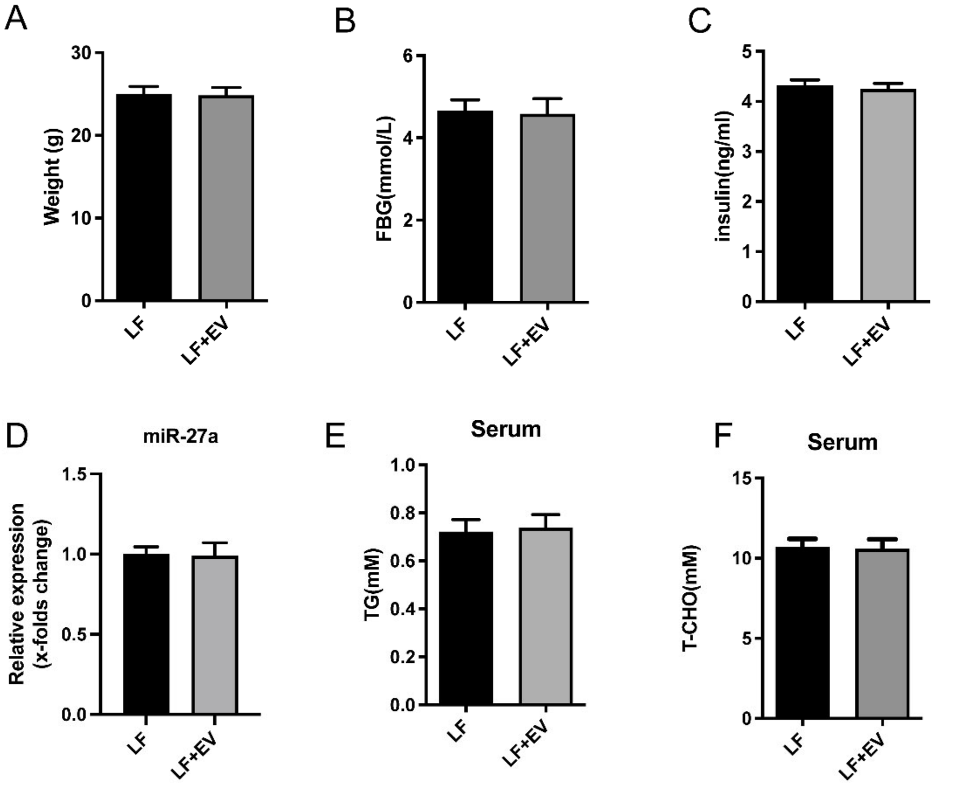

Figure S1 Lentiviral injection doesn't affect mouse parameters.

(A) Body weight, (B) FBG, (C) insulin, (D) serum miR-27a, (E) TG and (F) T-CHO levels in low-fat (LF) diet fed mice alone (LF) or in combination with lentiviral injection of empty vector (EV) after four weeks are shown . N=6, $p>0.05$,student's t-test.

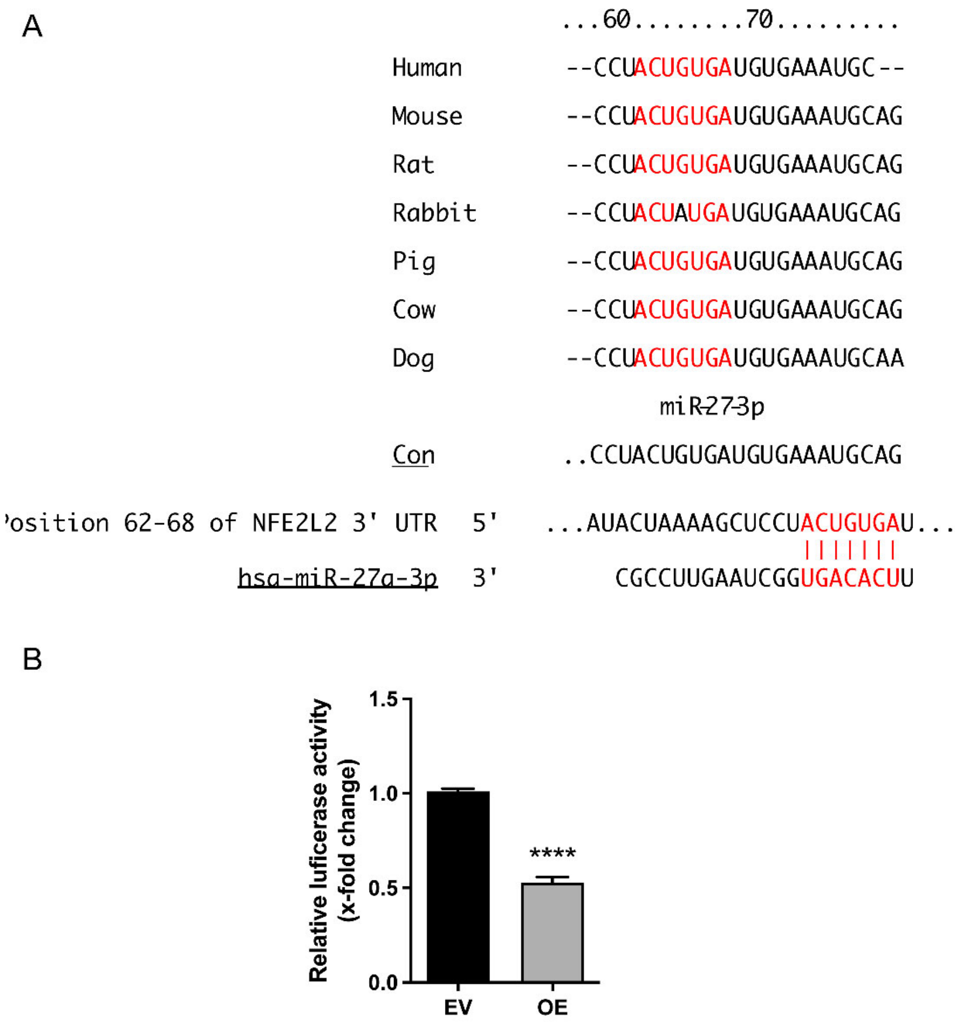

Figure S2 miR-27a binds at 3'UTR of NFE2L2.

(A) In silico prediction of miR-27a binds at 3'UTR of NFE2L2 by TargetScan is shown. (B) Luciferase reporter assay in cells treated with empty vector or miR-27a overexpression are shown. ****p<0.0001, student's t-test.
